# A Prediction Model for Optimal Primary Debulking Surgery Based on Preoperative Computed Tomography Scans and Clinical Factors in Patients With Advanced Ovarian Cancer: A Multicenter Retrospective Cohort Study

**DOI:** 10.3389/fonc.2020.611617

**Published:** 2021-01-07

**Authors:** Yu Gu, Meng Qin, Ying Jin, Jing Zuo, Ning Li, Ce Bian, Yu Zhang, Rong Li, Yu-mei Wu, Chun-yan Wang, Ke-qiang Zhang, Ying Yue, Ling-ying Wu, Ling-ya Pan

**Affiliations:** ^1^ Department of Obstetrics and Gynecology, Peking Union Medical College Hospital, Chinese Academy of Medical Sciences and Peking Union Medical College, Beijing, China; ^2^ Department of Obstetrics and Gynecology, National Cancer Center/Cancer Hospital, Chinese Academy of Medical Sciences and Peking Union Medical College, Beijing, China; ^3^ Department of Obstetrics and Gynecology, The West China Second University Hospital of Sichuan University, Chengdu, China; ^4^ Department of Obstetrics and Gynecology, Xiangya Hospital of Central South University, Changsha, China; ^5^ Department of Obstetrics and Gynecology, Chongqing University Cancer Hospital, Chongqing, China; ^6^ Department of Obstetrics and Gynecology, Beijing Obstetrics and Gynecology Hospital, Capital Medical University, Beijing, China; ^7^ Department of Obstetrics and Gynecology, Liaoning Cancer Hospital & Institute, Cancer Hospital of China Medical University, Shenyang, China; ^8^ Department of Obstetrics and Gynecology, Hunan Cancer Hospital, The Affiliated Cancer Hospital of Xiangya School of Medicine, Central South University, Changsha, China; ^9^ Department of Obstetrics and Gynecology, The First Hospital of Jilin University, Jilin, China

**Keywords:** ovarian cancer, computed tomography scans, prediction model, primary debulking surgery, neoadjuvant chemotherapy, multicenter study

## Abstract

**Objective:**

This study assessed the predictive value of preoperative computed tomography (CT) scans and clinical factors for optimal debulking surgery (ODS) in patients with advanced ovarian cancer (AOC).

**Methods:**

Patients with AOC in International Federation of Gynecology and Obstetrics (FIGO) stage III-IV who underwent primary debulking surgery (PDS) between 2016 and 2019 from nine tertiary Chinese hospitals were included. Large-volume ascites, diffuse peritoneal thickening, omental cake, retroperitoneal lymph node enlargement (RLNE) below and above the inferior mesenteric artery (IMA), and suspected pelvic bowel, abdominal bowel, liver surface, liver parenchyma and portal, spleen, diaphragm and pleural lesions were evaluated on CT. Preoperative factors included age, platelet count, and albumin and CA125 levels.

**Results:**

Overall, 296 patients were included, and 250 (84.5%) underwent ODS. The prediction model included age >60 years (*P*=0.016; prediction index value, PIV=1), a CA125 level >800 U/ml (*P*=0.033, PIV=1), abdominal bowel metastasis (*P*=0.034, PIV=1), spleen metastasis (*P*<0.001, PIV=2), diaphragmatic metastasis (*P*=0.014, PIV=2), and an RLNE above the IMA (*P*<0.001, PIV=2). This model had superior discrimination (AUC=0.788>0.750), and the Hosmer-Lemeshow test indicated its stable calibration (*P*=0.600>0.050). With the aim of maximizing the accuracy of prediction and minimizing the rate of inappropriate explorations, a total PIV ≥5 achieved the highest accuracy of 85.47% and identified patients who underwent suboptimal PDS with a specificity of 100%.

**Conclusions:**

We developed a prediction model based on two preoperative clinical factors and four radiological criteria to predict unsatisfactory debulking surgery in patients with AOC. The accuracy of this prediction model needs to be validated and adjusted in further multicenter prospective studies.

## Introduction

Epithelial ovarian cancer is currently the most malignant carcinoma of the female reproductive system with the highest mortality rate (1). Approximately two-thirds of ovarian cancer patients are initially diagnosed with advanced ovarian cancer (AOC) mainly due to the lack of early detection methods and specific symptoms for ovarian cancer (2). Primary debulking surgery (PDS) followed by platinum-based chemotherapy has been the standard treatment for patients with International Federation of Gynecology and Obstetrics (FIGO) stage IIIC or IV (3, 4) for many years. However, the traditional management of ovarian cancer has changed since two multicenter randomized phase III trials [EORTC 55971 (5) and CHORUS (6)] reported that neoadjuvant chemotherapy (NACT) followed by interval debulking surgery (IDS) was not inferior to PDS. NACT refers to chemotherapy as the primary treatment, which is administered to reduce the tumor burden before debulking surgery is performed (7, 8). The SCORPION (9) and JCOG0602 (10) clinical trials commonly considered that NACT-IDS was not inferior to PDS-CT due to less surgical aggressiveness and postoperative morbidity rates as well as better quality of life scores.

RD after debulking surgery is one of the most important independent risk factors for survival in AOC patients (11), regardless of surgery complexity or the administration of NACT (12, 13). Optimal debulking surgery (ODS) is considered optimal if the residual tumor (RD) is less than 1 cm in maximum diameter or thickness, which is associated with better survival outcomes than those achieved with suboptimal debulking surgery (SDS) (14, 15). Thus, improving the satisfactory cytoreduction rate and prolonging the overall survival of ovarian cancer patients are long-term goals for gynecological oncologists. The main value of NACT is reducing the tumor load and improving the feasibility of surgery and rate of ODS (16). However, many scholars have suggested that this benefit of surgery after NACT does not translate to a corresponding survival advantage and even increases the resistance of ovarian cancer patients to adjuvant chemotherapy (17–19). Two RCTs (5, 6) and some retrospective studies (20, 21) have shown no significant differences in survival outcomes between patients who receive NACT-IDS and those who receive PDS. Therefore, we explored a preoperative method of selecting the appropriate initial treatment option for AOC patients with the aim of achieving a high rate of ODS while avoiding excessive NACT (22).

Several researchers have explored specific preoperative detection methods for predicting ODS rates, including clinical factors, laboratory test results, radiological examination, laparoscopy evaluation and molecular features (23–28). Computed tomography (CT), as the most important and widely accepted imaging examination, is used to identify tumor distribution and a high tumor burden before performing cytoreduction (24). Recent studies have reported that the combination of clinical factors and CT could have a high accuracy of predicting ODS rates (29–32). The synthesized prediction system evaluates the correlation between clinical factors and CT findings to predict ODS rates and shows better sensitivity and specificity than clinical experience or limited screening methods. Several Western scholars have reported imaging-based models for predicting SDS rates in ovarian cancer patients (24, 29–36). However, it is difficult to apply and promote these models in various Chinese hospitals, especially in hospitals with doctors who have less experience in gynecological oncology. Although debulking surgery to reduce the sizes of residual tumors to the greatest extent possible should be the focus of cytoreductive efforts, complete resection is not feasible for all these hospitals due to the different levels of experience of gynecological teams. Therefore, it is necessary to develop an evidenced-based model to predict ODS for application in Chinese centers with multiple levels of experience.

We wanted to initially perform a retrospective study on a model development cohort and then perform a prospective study on a model validation cohort to make adjustments. We herein aimed to perform a multicenter retrospective cohort study to assess the values of preoperative CT and clinical factors and develop a scoring system for predicting the absence of ODS in patients with AOC undergoing primary surgery.

## Materials and Methods

### Study Population

Nine Chinese tertiary hospitals participated in this retrospective cohort study: Peking Union Medical College Hospital (PUMCH) as the main research center and eight other centers. Patients with FIGO stage III or IV ovarian, fallopian tube, or primary peritoneal cancer who underwent PDS between 09/01/2016 and 09/01/2019 were included. Patients with any of the following characteristics were excluded: 1) received neoadjuvant chemotherapy; 2) lacked critical clinical or operation data; and 3) underwent repeated collections. All patients provided written informed consent under approval by the ethics committee of their hospital. Patients who met the inclusion criteria were divided into two groups: 1) the ODS group: no gross RD and RD<1 cm in maximum tumor diameter and 2) the SDS group: RD≥1 cm in maximum tumor diameter.

### Data Collection

Key clinical data and CT image locations were recorded into unified electronic case report forms (CRFs) under an institutional review board-approved protocol and were obtained within 4 weeks before primary surgery. All contrast-enhanced CT scans were performed on modern technology conventional and spiral CT scanners, and were reviewed by the digital picture archiving system and at least two experienced radiologists. The preoperative clinical factors included the patient’s age, platelet count, albumin level, and CA125 level. The effect of age was shown to be independent of other variables, including the stage and grade (37). Compared to older patients, younger women with AOC have a survival advantage (38). In addition, increasing evidence indicates that the platelet count is a useful biomarker of long-term outcomes in patients with OC (39). The median OS was significantly decreased in patients with thrombocytosis or elevated CA125 levels (40). The following CT image locations were evaluated: 1) medium/large-volume ascites (defined as the presence of ascites on at least 2/3 of CT slices); 2) diffuse peritoneal thickening (defined as the presence of at least 2 separate peritoneal implants each>4 mm in size); 3) omental cake; 4) pelvic bowel metastasis≥1 cm (including the rectum and sigmoid colon); 5) abdominal bowel metastasis≥1 cm (including the small intestine as well as the ascending, transverse, and descending colon); 6) liver surface lesion (including the hepatorenal space); 7) liver parenchyma and portal lesion; 8) spleen metastasis (including the spleen surface and parenchyma); 9) diaphragmatic metastasis≥1 cm; 10) pleural metastasis≥1 cm; 11) retroperitoneal lymph node enlargement (RLNE) below the level of the inferior mesenteric artery (IMA)≥1 cm (including pelvic lymph nodes); 12) and RLNE above the level of the IMA≥1 cm (including the superior phrenic lymph node). The data used to support the findings of this study are available from the corresponding author upon request.

### Statistical Analysis

All statistical analyses were performed using SPSS software (version 23.0; SPSS Inc., Chicago, IL, USA). Student’s t-tests and Mann-Whitney U tests were used to compare continuous variables. Pearson’s chi-squared tests and Fisher’s exact tests were used to compare categorical variables. Continuous variables with a normal distribution are presented as the means ± standard deviations (SDs), and nonnormally distributed variables are reported as the medians ± interquartile ranges (IQRs) (41). Each of the clinical factors and radiological criteria were individually evaluated by using logistic regression for the univariate analysis. Then, all the variables with significant differences based on the univariate analysis were calculated by binary logistic regression with stepwise forward selection. The associations were evaluated by odds ratios (ORs) and corresponding 95% confidence intervals (CIs). Then, a prediction model was constructed using the variables with significant differences based on the multivariate analysis, and the prediction index value (PIV) was systematically calculated by the weight of each variable and corresponding functions. Statistical significance was set at P<0.050.

The receiver operating characteristic (ROC) curve was drawn to evaluate the discrimination performance of the prediction model. The area under the curve (AUC) and 95% CI were determined to assess its prediction ability. The Hosmer-Lemeshow (H-L) goodness of fit test was used to evaluate the calibration performance of the prediction model (41). The sensitivity, specificity, positive predictive value (PPV), negative predictive value (NPV), and accuracy of each PIV were calculated according to different cut-off values. Sensitivity was defined as the number of patients receiving SDS and were correctly identified (true positives) divided by the total number of patients receiving SDS (true positives + false negatives). Specificity was defined as the number of patients receiving ODS who were correctly identified (true negatives) divided by the total number of patients receiving ODS (true negatives + false positives). The PPV was calculated as the number of true positives divided by the total number of positive results (true positives + false positives), and the NPV was defined as the number of true negatives divided by the total number of negative results (true negatives + false negatives). Accuracy was calculated as the number of true positives plus true negatives (total number correct) divided by the total number of patients studied (42). Youden’s index (or the C-index), which indicates the maximum potential effectiveness of a biomarker, is a common summary measure of the ROC curve (43). We used Youden’s index to determine the cut-off PIV point that yielded the maximum sensitivity and specificity for predicting suboptimal cytoreduction.

## Results

### Flow Diagram and Characteristics of Included Patients

**Figure S1** shows the flow chart of this study population. A total of 303 patients from nine centers were enrolled. After excluding five patients without critical information and two duplicate patients, a total of 296 AOC patients who met the inclusion criteria were ultimately included. A total of 250 patients were in the ODS group (including 163 patients with no RD and 87 patients with an RD of 0-1 cm), and 46 patients were in the SDS group. We kept the power of the study maximum. **Table 1** shows the preoperative clinical factors and radiological criteria of the included ovarian cancer patients. The mean patient age was 53.5 (± 10.5) years in the ODS group and 55.7 (± 12.8) years in the SDS group. There was no significant difference in the perioperative platelet count (*P*=0.781) or perioperative albumin level (*P*=0.869) between the two groups. The most common abnormalities observed on CT images were omental cake (53.4%), diffuse peritoneal thickening (38.9%), and pelvic bowel metastasis (34.5%). For most of the radiological criteria, there were significant differences between the two groups, thus indicating that the comparison of these variables was meaningful.

**Table 1:** The preoperative clinical factors and radiological criteria and of included ovarian cancer patients.

Variables	ODS group(N = 250)	SDS group(N = 46)	*P*
**Preoperative clinical factor**			
**Age**	53.5 ± 10.5	55.7 ± 12.8	0.017
≤60 years	180 (72.0)	25 (54.3)	
>60 years	70 (28.0)	21 (45.7)	
**Perioperative CA125**	472.8 ± 650.6	573.2 ± 1052.6	0.021
≤800 U/ml	188 (75.2)	27 (58.7)	
>800 U/ml	62 (24.8)	19 (41.3)	
**Perioperative platelet**	255.9 ± 0	255.9 ± 1.5	0.781
≤350 (10^9^/L)	221 (88.4)	40 (87.0)	
>350 (10^9^/L)	29 (11.6)	6 (13.0)	
**Perioperative albumin**	44.2 ± 3.8	44.0 ± 2.7	0.869
≤35 g/L	18 (7.2)	3 (6.5)	
>35 g/L	232 (92.8)	43 (93.5)	
**Radiological criterion**			
**Median-Large volume ascites**	64 (25.6)	18 (39.1)	0.059
**Diffuse peritoneal thickening**	91 (36.4)	24 (52.2)	0.044
**Omental cake**	125 (50.0)	33 (71.7)	0.007
**Pelvic bowel metastasis**	78 (31.2)	24 (52.2)	0.006
**Abdominal bowel metastasis**	33 (13.2)	13 (28.3)	0.010
**Liver surface lesion**	39 (15.6)	20 (43.5)	<0.001
**Liver parenchyma and portal lesion**	13 (5.2)	8 (17.4)	0.003
**Spleen metastasis**	19 (7.6)	14 (30.4)	<0.001
**Diaphragmatic metastasis**	3 (1.2)	4 (8.7)	0.002
**Pleural metastasis**	4 (1.6)	2 (4.3)	0.224
**RLNE below the level of IMA**	65 (26.0)	18 (39.1)	0.068
**RLNE above the level of IMA**	32 (12.8)	16 (34.8)	<0.001

Data are presented as number (%) or mean (± SD) or median (± IQR). ODS, Optimal debulking surgery; SDS, Suboptimal debulking surgery; RLNE, Retroperitoneal lymph nodes enlargement; IMA, Inferior mesenteric artery.

### Assessment of the Prediction Model

**Table 2** shows the univariate analysis of the included ovarian cancer patients. In the univariate analysis, two clinical factors were associated with suboptimal cytoreduction: age>60 years (P=0.019) and perioperative CA125 level>800 U/ml (P=0.023). Nine radiological criteria were related to suboptimal cytoreduction: diffuse peritoneal thickening (P=0.046), omental cake (P=0.008), pelvic bowel metastasis (P=0.007), abdominal bowel metastasis (P=0.012), liver surface lesion (P<0.001), liver parenchyma and portal lesion (P=0.005), spleen metastasis (P<0.001), diaphragmatic metastasis (P=0.008), and RLNE above the level of the IMA (P<0.001). Then, we performed a multivariate analysis based on the significant factors from the univariate analysis. We further calculated the PIV, which was assigned based on the multivariate regression coefficient and OR. **Table 3** shows the prediction model of all significant clinical and radiological criteria based on the multivariate analysis for ODS. The predictive index parameters related to a high risk of successful SDS included age>60 years (*P*=0.016, PIV=1), CA125 level>800 U/ml (*P*=0.033, PIV=1), abdominal bowel metastasis (*P*=0.034, PIV=1), spleen metastasis (*P*<0.001, PIV=2), diaphragmatic metastasis (*P*=0.014, PIV=2), and RLNE above the level of the IMA (*P*<0.001, PIV=2).

**Table 2:** The univariate analysis of included ovarian cancer patients.

Variables	N	OR	95%CI	*P*
**Age**				0.019
≤60 years	205	1		
>60 years	91	2.160	1.136–4.107	
**Perioperative CA125**				
≤800 U/ml	215	1		0.023
>800 U/ml	81	2.134	1.110–4.101	
**Perioperative platelet**				0.781
≤350 (10^9^/L)	261	1		
>350 (10^9^/L)	35	1.143	0.446–2.930	
**Perioperative albumin**				0.869
>35 g/L	275	1		
≤35 g/L	21	0.899	0.254–3.185	
**Median-Large volume ascites**				
No	214	1		0.062
Yes	82	0.535	0.278–1.032	
**Diffuse peritoneal thickening**				0.046
No	181	1		
Yes	115	1.906	1.012–3.591	
**Omental cake**				0.008
No	138	1		
Yes	158	2.538	1.276–5.051	
**Pelvic bowel metastasis**				0.007
No	194	1		
Yes	102	2.406	1.272–4.550	
**Abdominal bowel metastasis**				0.012
No	250	1		
Yes	46	2.590	1.237–5.424	
**Liver surface lesion**				<0.001
No	237	1		
Yes	59	4.162	2.118–8.179	
**Liver parenchyma and portal lesion**				0.005
No	275	1		
Yes	21	3.838	1.492–9.874	
**Spleen metastasis**				<0.001
No	263	1		
Yes	33	5.319	2.431–11.639	
**Diaphragmatic metastasis**				0.008
No	289	1		
Yes	7	7.841	1.694–36.295	
**Pleural metastasis**				0.243
No	290	1		
Yes	6	2.795	0.497–15.728	
**RLNE below the level of IMA**				0.071
No	213	1		
Yes	83	1.830	0.949–3.526	
**RLNE above the level of IMA**				<0.001
No	248	1		
Yes	48	3.633	1.784–7.399	

N, number; OR, Odds ratio; RLNE, Retroperitoneal lymph nodes enlargement; IMA, Inferior mesenteric artery.

**Table 3:** The model of significant clinical and radiological criteria based on multivariate analysis for predicting suboptimal debulking surgery.

Predictive index parameter	N	RC	OR	95%CI	*P*	PIV
**Preoperative clinical factor**						
**Age**		0.875			0.016	
≤60 years	205		1			0
>60 years	91		2.399	1.179–4.883		1
**Perioperative CA125**		0.799			0.033	
≤800 U/ml	215		1			0
>800 U/ml	81		2.223	1.067–4.631		1
**Radiological criterion**						
**Abdominal bowel metastasis**		0.888			0.034	
No	250		1			0
Yes	46		2.430	1.070–5.516		1
**Spleen metastasis**		1.546			<0.001	
No	263		1			0
Yes	33		4.692	1.987–11.077		2
**Diaphragmatic metastasis**		2.116			0.014	
No	289		1			0
Yes	7		8.300	1.537–44.818		2
**RLNE above the level of IMA**		1.570			<0.001	
No	248		1			0
Yes	48		4.808	2.176–10.623		2

N, number; RC, Regression coefficient; OR, Odds ratio; PIV, Prediction index value; RLNE, Retroperitoneal lymph nodes enlargement; IMA, Inferior mesenteric artery.

### Evaluation of the Prediction Model

**Figure 1:**
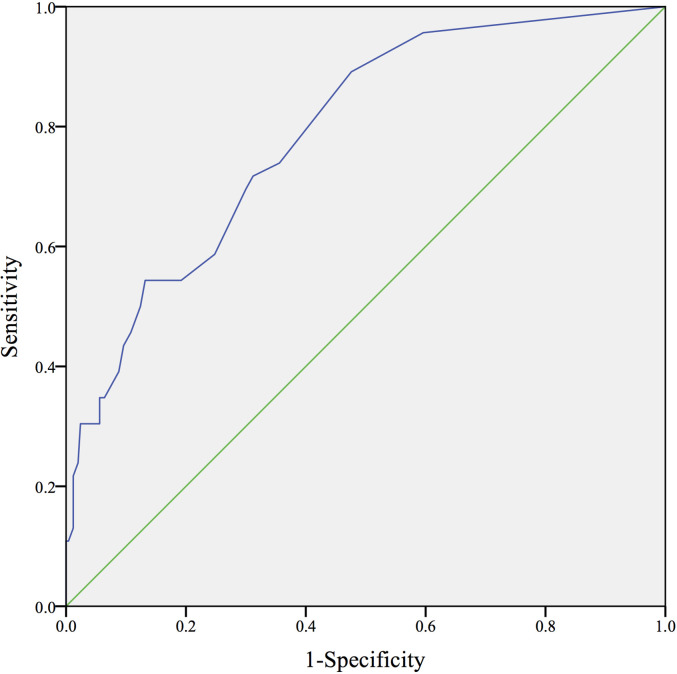
The ROC curve of the prediction model. (AUC=0.788, 95%CI=0.720-0.856).

**Figure 1** shows the ROC curve of the prediction model. The AUC was 0.788 (greater than 0.750), showing that this prediction model has superior discrimination. In addition, there was no significant difference between the predictive value and real value in the Hosmer-Lemeshow test (X^2^ = 2.752, *P*=0.600>0.050), which indicated the stable calibration of the prediction model. **Table 4** shows the overall prediction model according to different cut-off values. The PIV ranged from 0 to nine points. The sensitivity, specificity, PPV, NPV, and overall accuracy of each PIV were determined. Youden’s index of the ROC curve was 41.5%, which corresponded to a sensitivity of 89.1% and a specificity of 52.4%. Consequently, with the aim of maximizing the accuracy of prediction and minimizing the rate of inappropriate explorations, a PIV of ≥5 achieved the highest accuracy of 85.47% and identified patients who underwent SDS with a specificity of 100%.

**Table 4:** The overall prediction model according to different cut-off values.

PIV	Sensitivity (%)	Specificity (%)	PPV (%)	NPV (%)	Accuracy (%)
≥0	100.00	0.00	15.54	N/A	15.54
≥1	95.65	40.40	22.80	98.06	48.99
≥2	71.74	68.80	29.73	92.97	69.26
≥3	54.35	86.80	43.10	91.18	81.76
≥4	30.43	94.80	51.85	88.10	84.80
≥5	6.52	100.00	100.00	85.32	85.47
≥6	2.17	100.00	100.00	84.75	84.80
≥7	2.17	100.00	100.00	84.75	84.80
≥8	0.00	100.00	100.00	84.46	84.80
≥9	0.00	100.00	100.00	84.46	84.80

PIV, prediction index value; PPV, positive predictive value; NPV, negative predictive value; N/A, not applicable.

## Discussion

At present, NACT-IDS is an alternative treatment option for AOC patients who may initially have a low incidence of ODS (7, 8). However, there is no unified standard method of selecting appropriate patients or determining the best time to perform PDS. Some gynecologists reported that the following clinical factors were associated with a high rate of unsatisfactory cytoreduction: 1) extensive implant metastasis in the upper abdomen or thorax and a large tumor burden throughout the whole body and 2) a poor performance status that could not tolerate cytoreduction (e.g., advanced age, high-risk complications, or the combination of a large amount of ascites and hydrothorax) (16, 44). However, this type of clinical report lacks scientific evidence and cannot be widely accepted and applied by different gynecologists in various hospitals. Therefore, the selection of appropriate AOC patients for undergoing primary surgery to obtain the highest success rate of ODS has been a focus in the past few decades.

Laparoscopy can be used to assess the probability of having no residual tumor before PDS and to evaluate tumor size, the degree of tumor spread, and tumor infiltration in surrounding tissues (26, 27, 45). Fagotti et al. developed a widely accepted staging laparoscopy scoring system for determining candidates for primary surgery (46) that includes omental cake (PIV=1), extensive peritoneal (PIV=1) and diaphragmatic (PIV=1) carcinosis, mesenteric retraction (PIV=2), bowel (PIV=2) and stomach (PIV=2) infiltration, and superficial spleen and/or liver metastasis (PIV=2). At a PIV≥8, the probability of ODS during laparotomy is equal to 0; thus, NACT is initially recommended. This laparoscopy model can avoid an unnecessary exploratory laparotomy and improve individualized treatment for AOC patients. However, as an invasive manipulation procedure, laparoscopy has the risks of anesthesia and surgical complications (11). In addition, the evaluation of lesions under retroperitoneal and retrohepatic areas is limited (47). Furthermore, we must consider the cost-effectiveness benefit: laparoscopy is more time consuming and expensive than other preoperative examinations (47). Due to the above limitations, it is difficult to perform a staging laparoscopy before PDS in most Chinese hospitals. In addition, the molecular features played important roles in patients with OC, affecting the status of the BRCA gene and homologous recombination deficiency (HRD) (28, 48). However, primary targeted therapy for OC lacks sufficient evidence, and most patients cannot afford the high cost of gene detection. Therefore, we aimed to develop a preoperative evaluation model that combines clinical features with imaging examination features to allow patients to obtain the greatest benefit.

The model used to predict the rate of unsatisfactory debulking surgery in our study ultimately included two preoperative clinical factors and four radiological criteria based on the multivariate analysis of all enrolled patients. The two clinical factors, age>60 years and CA125 level>800 U/ml, indicated that elderly patients who have a high tumor burden and a poor general condition are not suitable for PDS. The four radiological criteria showed that tumors located in the upper abdomen or thorax, such as the diaphragm, spleen, and upper retroperitoneal lymph nodes, greatly influence the cytoreduction rate, as they are truly difficult to remove in debulking surgery. The evaluation of retroperitoneal disease extension plays a very important prognostic role for patients with OC (49, 50). Our prediction model is a scoring system; therefore, the difficulty of PDS and the rate of having no RD are not determined by a single high-risk factor but rather by the accumulation degree of high-risk factors. This may be attributed to poor surgical tolerance, a long surgical time, and surgical complications during PDS if an AOC patient has a high score in our prediction model. Therefore, our model is basically consistent with the experience of gynecological oncologists (44, 51) but has more scientific- and evidence-based support.

Several scholars have published imaging-based models for predicting SDS rates in ovarian cancer patients (24, 29–31, 33–36, 52); these are summarized in **Table 5**. Among them, the model from Suidan et al. at Memorial Sloan-Kettering Cancer Center (MSKCC) is one of the most common and high evidence-based imaging models (29). However, in our practical application, it was found that the imaging classification of this model is excessively detailed and complex and often requires professional senior imaging doctors to read CT scans. It is difficult for gynecologists at different levels to apply this model, and there is also great difficulty in promoting this model in Chinese hospitals. Therefore, we initially roughly divided the body into several large areas based on our previous surgical experience (e.g., the pelvic and abdominal bowels, spleen, liver, and RLN). Our model may be more operable and applicable than the MSKCC model for gynecologic oncologists in various Chinese hospitals. In addition, our prediction model has a specificity of 100% and an accuracy of 85.47%, which shows superior discrimination and stable calibration.

**Table 5:** The model comparison of predicting suboptimal debulking surgery based on radiological criteria in ovarian cancer from published reports.

Study	Types	Clinical factors	CT factors	Model ability
**Axtell et al. (** 33 **)**	Multi-Institutional Reciprocal Validation Study (UCLA, et al.)	–	①Diaphragm disease ②large bowel mesentery implants	A sensitivity of 79%, a specificity of 75%, and an accuracy of 77%.
**Ferrandina et al. (** 31 **)**	Retrospective, single center (Italy)	①Age ②CA-125 ③ECOG-PS	①DPT ②Peritoneal implants>2 cm ③Bowel mesentery involvement ④Omental cake ⑤Pelvic sidewall involvement and/or hydroureter ⑥Suprarenal aortic lymph nodes>1cm ⑦ Infrarenal aortic lymph nodes>2 cm ⑧Superficial liver metastases>2 cm and/or intraparenchimal liver metastases any size ⑨Large volume ascites	Specificity>75%, PPV and NPV>50%, Accuracy>60%
**Stashwick et al. (** 29 **)**	Retrospective, single center (Denver)	①Albumin<2.7 (PIV=1) ②CA-125≥500 (PIV=1)	①Bowel mesentery involvement>2 cm (PIV=1) ②Diffuse peritoneal studding (PIV=2) ③Para-aortic lymphadenopathy>2 cm(PIV=1) ④Splenic disease>1 cm (PIV=1)	The sensitivity, specificity, PPV, and NPV were 94%, 75%, 80%, and 93%.
**Fujwara et al. (** 35 **)**	Retrospective, single center (Osaka)	–	Both: ①DPT ②Infrarenal para-aortic or pelvic lymph node ③Bowel encasement tumor≥2 cm ④Any tumor implants in the cul-de-sac Model 1: Adds consideration to any tumors in the pelvic or retroperitoneum Model 2: ①Bowel mesenteries≥2 cm ②Omental caking≥2 cm) ③Ascites fluid	Model 1: accuracy of 90.8%Model 2: accuracy of 93.9%
**Kim et al. (** 34 **)**	Retrospective, single center (Iksan)	–	①Omental extension to the stomach or spleen ②Inguinal or pelvic lymph nodes	A PPV of 100%, a specificity of 100%, and an accuracy of 45.8%.
**Shim et al. (** 30 **)**	Retrospective, single center (Seoul)	Surgical aggressive index	①Diaphragm disease ②Ascites ③Peritoneal carcinomatosis ④Small bowel mesentery implant ⑤Tumoral uptake ratio	A predictive accuracy of 88.1%.
**Janco et al. (** 36 **)**	Retrospective, single center (Mayo Clinic)	①ECOG performance status ≥2 (OR=5.13)	①DPT (OR=3.94) ②Lymphadenopathy (OR=3.00)	A sensitivity of 23.1% and specificity of 94.1%.
**Nasser et al. (** 24 **)**	Retrospective, single center (London)	–	①Diaphragmatic ②Spleen ③Large bowel ④Small bowel ⑤Rectum ⑥Porta hepatis ⑦Mesenteric disease ⑧Lymph node	A high specificity of 65% but low sensitivity.
**Suidan et al. (** 28 **)**	Prospective, non-randomized, multicenter (MSKCC, et al.)	①Age ≥ 60 years (OR= 1.5) ②CA-125≥600 U/mL (OR = 1.3) ③ASA 3–4 (OR= 1.6);	①SMA (OR=4.1) ②Splenic hilum/ligaments (OR= 1.4) ③Lesser sac>1cm (OR=2.2) ④Gastrohepatic ligament/porta hepatis (OR=1.4) ⑤Gallbladder fossa/intersegmental fissure (OR=2) ⑥Suprarenal RLN (OR=1.3) ⑦Small bowel (OR=1.1) ⑧Moderate-severe ascites (OR=2.2).	When a predictive score was 0–2, 3–5, 6–8, and ≥9, the predictive SDS rate was 45%, 68%, 87%, and 96%.
**Llueca et al. (** 52 **)**	Retrospective, single center		①Lung metastasiser②Hepatic metastasis in 3 or more san③hepatic segments ④Severe hepatic pedicle involvement ⑤Progression after NACT ⑥Diffuse serous small bowel disease	A sensitivity of 83% (R4 model) and 69% (R3 model).

CT, Computed tomography scan; PIV, prediction index value; PPV, positive predictive value; NPV, negative predictive value; DPT, Diffuse peritoneal thickening; OR, Odds ratio; PIV, Prediction index value; MSKCC, Memorial Sloan-Kettering Cancer Center; SMA, Superior mesenteric artery; RLN, Retroperitoneal lymph nodes; SDS, Suboptimal debulking surgery.

On the other hand, Llueca et al. proposed a predictive model with the peritoneal cancer index (PCI), which can provide more detailed information about peritoneal spread. The PCI is used to quantitatively assess cancer distribution in the peritoneum based on the sizes of lesions in 13 abdominopelvic regions, and patients were classified into three categories with scores of 1–10, 11–20, and >20 (53). In the pilot study, their models predicted suboptimal or complete and optimal cytoreductive surgery with sensitivities of 83% (R4 model) and 69% (R3 model). A PCI>20 was a major risk factor for unresectability in patients with AOC (52). In our study, peritoneal metastasis was evaluated in the variable statistics but was not included in the final multivariate model. PCI is a preferable supplement to our model, which could be improved by including this assessment of the PCI. In addition, several studies reported that minimally invasive interval debulking surgery (MI-IDS) played a positive role in the quality of life and surgery complications (27, 54). However, the range of surgical resection in MI-IDS is limited, and a thorough peritoneal evaluation is not possible. It is difficult to reach ODS, resulting in residual tumors and worsened oncologic outcomes. MI-IDS may considered be limited to low-complexity standard debulking surgery (55, 56).

Moreover, the greatest advantage of this study is that it was a multicenter study that collected patient data from nine large tertiary hospitals in China. The large-scale study, wide area coverage, and high quantity of enrolled patients ensured the diversity and credibility of this report. This retrospective study is not only the basis for creating a predictive model but can also be further verified in subsequent multicenter prospective studies. After modification and identification, it may ultimately be promoted in most hospitals in China, thus achieving the purpose of this whole study.

There are several limitations to this study. First, due to the natural limitations of retrospective research, unknown potential confounders and selection biases may be present (57). However, we attempted to make a unified standard for collecting patients and evaluating CT sites to ensure that all data were collected in a similar manner. Moreover, we balanced the confounding factors between the two groups by Cox multivariate regression analysis when there were a few heterogeneities in baseline factors between the ODS and SDS groups. Second, the horizontal differentiation among multiple centers may lead to data deviations. The centers participating in this study were all high-quality large-scale hospitals in China, and gynecologists had sufficient experience in the diagnosis and treatment of ovarian cancer. The inclusion criteria for our study population and data collection were unified standards. The capabilities of performing debulking surgery and reading CT scans were similar among the various centers. Currently, a multicenter, nonrandom prospective study is ongoing. We expect this prediction model to be verified and adjusted by collecting additional data.

## Conclusion

In conclusion, we developed a prediction model based on two preoperative clinical factors and four radiological criteria for predicting unsatisfactory debulking surgery in AOC patients: included age>60 years (PIV=1), CA125 level>800 U/ml (PIV=1), abdominal bowel metastasis (PIV=1), spleen metastasis (PIV=2), diaphragmatic metastasis (PIV=2), and RLNE above the level of the IMA (PIV=2). A total PIV of ≥5 in this model may indicate a high risk associated with undergoing SDS, with an accuracy of 85.47% and a specificity of 100%. The accuracy of this prediction model needs to be validated and adjusted in further multicenter prospective studies. We look forward to collecting additional information from ovarian cancer patients and learning from research experience at other centers.

## Data Availability Statement

The raw data supporting the conclusions of this article will be made available by the authors, without undue reservation.

## Ethics Statement

The studies involving human participants were reviewed and approved by the ethics committee of each hospital. In the main center, all patients provided written informed consent under approval by the ethics committee of Peking Union Medical College Hospital.

## Author Contributions

L-YW and L-YP conceptualized the study. YG, MQ, and YJ contributed to the methodology. YJ, JZ, and NL validated the study. YG, MQ, and YJ contributed to the formal analysis and investigation. YG and MQ wrote and prepared the original draft. All authors wrote and prepared the original draft. L-YW and L-YP acquired the funding. All authors provided the resources. L-YW and L-YP supervised the study. All authors contributed to the article and approved the submitted version.

## Funding

This project was supported by The Fund of The National Key R&D Program of China 2016YFC1303700 (Affiliated project 2016YFC1303701, 2016.9-2020.12). Furthermore, this project was also supported by CAMS Innovation Fund for Medical Sciences (CIFMS-2017-I2M-1-002, 2017.01-2020.12).

## Conflict of Interest

The authors declare that the research was conducted in the absence of any commercial or financial relationships that could be construed as a potential conflict of interest.
