# Supplementary material for: A Prediction Model for Optimal Primary Debulking Surgery Based on Preoperative Computed Tomography Scans and Clinical Factors in Patients With Advanced Ovarian Cancer: A Multicenter Retrospective Cohort Study
Source: Front Oncol. 2021 Jan 7;10:611617. doi: 10.3389/fonc.2020.611617 (PMC7819136; doi:10.3389/fonc.2020.611617)
Supplement: Supplementary file 1 [file DataSheet_1.doc]

**Supplementary materials**

**Figure S1. The flow diagram of the study population. (**Abbreviations: AOC=Advanced ovarian patients; RD=Residual disease.)
